# A Preliminary Study of the Influence of Age of Onset and Childhood Trauma on Cortical Thickness in Major Depressive Disorder

**DOI:** 10.1155/2014/410472

**Published:** 2014-03-06

**Authors:** Natalia Jaworska, Frank P. MacMaster, Ismael Gaxiola, Filomeno Cortese, Bradley Goodyear, Rajamannar Ramasubbu

**Affiliations:** ^1^Mathison Centre for Mental Health Research & Education, Department of Psychiatry, University of Calgary, TRW Building, 3280 Hospital Drive NW, Calgary, AB, Canada T2N 4Z6; ^2^Hotchkiss Brain Institute, University of Calgary, 3330 Hospital Drive NW, Calgary, AB, Canada T2N 4N1; ^3^Alberta Children's Hospital Research Institute, University of Calgary, 2888 Shaganappi Trail NW, Calgary, AB, Canada T3B 6A8; ^4^Department of Pediatrics, University of Calgary, 2888 Shaganappi Trail NW, Calgary, Canada T3B 6A8; ^5^The Seaman Family Centre, University of Calgary, Foothills Medical Centre, 1403-29th Street NW, Calgary, AB, Canada T2N 2T9; ^6^Department of Clinical Neurosciences, University of Calgary, Foothills Medical Centre, 1403-29th Street NW, Calgary, AB, Canada T2N 2T9

## Abstract

*Background*. Major depressive disorder (MDD) neural underpinnings may differ based on onset age and childhood trauma. We assessed cortical thickness in patients who differed in age of MDD onset and examined trauma history influence. *Methods*. Adults with MDD (*N* = 36) and controls (HC; *N* = 18) underwent magnetic resonance imaging. Twenty patients had MDD onset <24 years of age (pediatric onset) and 16 had onset >25 years of age (adult onset). The MDD group was also subdivided into those with (*N* = 12) and without (*N* = 19) physical and/or sexual abuse as assessed by the Childhood Trauma Questionnaire (CTQ). Cortical thickness was analyzed with FreeSurfer software. *Results*. Thicker frontal pole and a tendency for thinner transverse temporal cortices existed in MDD. The former was driven by the pediatric onset group and abuse history (independently), particularly in the right frontal pole. Inverse correlations existed between CTQ scores and frontal pole cortex thickness. A similar inverse relation existed with left inferior and right superior parietal cortex thickness. The superior temporal cortex tended to be thinner in pediatric versus adult onset groups with childhood abuse. *Conclusions*. This preliminary work suggests neural differences between pediatric and adult MDD onset. Trauma history also contributes to cytoarchitectural modulation. Thickened frontal pole cortices as a compensatory mechanism in MDD warrant evaluation.

## 1. Introduction

Major depressive disorder (MDD) is a common psychiatric disorder with a high burden of disease, yet its neural underpinnings remain elusive. A handful of studies have assessed spatial patterns of cortical thickness in MDD. Interestingly, the regional patterns of cortical thinning in MDD do not perfectly reflect what would be expected from the neuroimaging literature (i.e., cortical thinning is not confined to cognitive and emotive centers; fronto-cortico-limbic structures) [1]. Further, extant literature is not consistent with respect to which cortical regions are typically thicker/thinner in the disorder. This indicates a need for further study with careful attention to factors that may influence cortical thickness in MDD, such as age of disorder onset and past trauma and neglect.

The majority of research on cortical thickness in MDD has focused on assessing elderly depressed individuals (typically defined as >60 years of age; late-life MDD). For instance, one group found no cortical thickness differences between older depressed females and controls [2]. Similarly, Colloby et al. [3] noted no cortical thickness differences in frontal lobe structures between older individuals with MDD and controls. However, they found a tendency for decreased cortical thickness in MDD in the left frontal pole/pars orbitalis and the right medial orbitofrontal region. In yet another study, Kumar et al. [4] noted a thinner right isthmus in an elderly depressed cohort compared with controls. Another group found that elderly depressed individuals demonstrated thinner cortices in frontal (medial/superior), superior parietal, and inferior temporal regions [5]. Further, treatment nonresponders (versus responders) demonstrated thinner cortices in bilateral posterior cingulate and parahippocampal regions, the left paracentral, pre/cuneus and insular cortices as well as the right medial orbitofrontal, lateral occipital, and superior postcentral cortices [5]. Yet others reported a thinner bilateral dorsolateral prefrontal cortex (DLPFC) and thinner postcentral region in elderly depressed individuals relative to controls. Cortical thinning was also found in the left prefrontal (orbitofrontal, pars triangularis), rostral anterior cingulate, medial/superior temporal, and parietal cortices as well as in the pre/paracentral gyri. Right hemisphere thinning was noted in the pars opercularis, rostral middle frontal, precuneus, and isthmus cortices in elderly individuals with MDD [6]. Finally, cortical thickness in the frontal pole, superior/middle frontal gyrus, orbitofrontal gyrus, and anterior cingulate gyrus was thinner in elderly depressed patients relative to controls [7]. In sum, while some have noted no cortical thickness differences in elderly depressed versus control individuals, others have. Research points to decreased cortical thickness in prefrontal regions, particularly in the orbitofrontal area, in superior/middle frontal aspects (including the DLPFC) as well as para/postcentral regions and the cuneus/isthmus regions in elderly individuals with MDD.

Assessments of cortical thickness in nonelderly adults (i.e., those younger than 60 years of age) with MDD are sparse. Järnum et al. [8] found thinner cortices in MDD patients (middleaged) compared with controls in the orbitofrontal cortex, superior temporal lobe, and insula. Further, depressed nonremitters exhibited a thinner posterior cingulate cortex compared to those in remission. Similarly, another group noted thinner cortices in nonelderly (18–60 years of age) individuals with MDD in the left parahippocampal gyrus, orbitofrontal cortex as well as in the right middle/superior frontal gyri (DLPFC), middle temporal gyrus, and insula [9]. Yet another group noted that depressed adults showed cortical thinning in the bilateral superior/middle frontal gyri, right precentral gyrus (i.e., DLPFC), and right orbitofrontal gyrus. Smaller clusters of cortical thinning existed in the parietal (bilateral inferior parietal regions and left post-central gyrus), temporal (left entorhinal and middle temporal cortex), and occipital lobes (left lateral occipital and lingual gyrus). Regions that were thicker in MDD were the left anterior insula and lateral orbitofrontal gyrus [10]. Recently, van Eijndhoven et al. [11] assessed medication-naïve patients during their first major depressive episode (MDE) or after their first MDE. The medial orbitofrontal cortex was thinner in the MDD patients than in controls. Conversely, the temporal pole and the caudal anterior and posterior cingulate cortices were thicker. This was evident in both currently depressed and recovered patients, suggesting trait-versus state-specific abnormalities. Thus, assessments of nonelderly adults with MDD suggest cortical thinning in the medial orbitofrontal cortex (though lateral regions may be associated with thickening), insula, DLPFC, and the middle temporal cortex—which partially overlap with findings in elderly depressed individuals.

Finally, a handful of groups have assessed cortical thickness in pediatric MDD and found thinner cortices in the right pericalcarine, postcentral, and superior parietal gyri as well as the left supramarginal gyrus. The pediatric MDD cohort (≤18 years of age) exhibited thicker bilateral temporal pole cortices [12], consistent with the results in adults [11]. Additionally, our group observed thicker bilateral middle frontal gyri and left caudal cingulate gyrus in MDD adolescents compared to controls [13].

Potential factors that may contribute to the inconsistency in cortical thickness findings in MDD include the age of the sample examined, medication status, illness severity, sex, and age of MDD onset. The latter is perhaps the most pertinent as later childhood/adolescence is marked by extensive brain changes [14–16]. As such, early MDD onset (i.e., pediatric/adolescent onset) may interfere with normal neurodevelopmental trajectories and manifest as structural abnormalities in adulthood. Further, early MDD onset appears to be associated with increased risk for disorder recurrence, illness burden, and psychiatric comorbidities [14]. This suggests that early MDD onset may be associated with specific neurobiological features. However, few studies have assessed the effect of age of onset on cortical thickness in MDD. A recent study examined the association between age of MDD onset (in this case, early: <24 years; late: >25 years) and cortical thickness [1]. Reductions were found in the DLPFC, pre/postcentral gyri and the lingual gyrus in the early MDD onset group versus controls. Further analyses revealed thicker cortices in the early versus late MDD onset groups in the bilateral posterior cingulate cortex. Conversely, the left parahippocampal, right lingual, right fusiform, and right precuneus gyri were thinner in the early versus the late onset MDD group. Another group assessed elderly depressed patients with earlier (<60 years) and late-life (>60 years) MDD onset and found that the left anterior cingulate was thinner in the late-life onset group [17]. Though preliminary, such data suggest that age of onset may play a role in the spatial distribution of cortical thickness findings in MDD.

Early adverse events increase the possibility of MDD development later in life [18]. Early trauma/maltreatment may interfere with normal brain development. Previous work has reported cortical thickness reductions in maltreated versus nonmaltreated children in the anterior cingulate, superior frontal gyrus, orbitofrontal cortex, left middle temporal regions, and lingual gyrus [19]. Heim et al. [20] also reported widespread cortical thinning as a function of childhood adversity (assessed by the Childhood Trauma Questionnaire (CTQ)). CTQ scores were specifically associated with anterior cingulate gyrus, precuneus, and parahippocampal gyrus cortical thinning. These studies parallel morphometric work that has noted grey matter density and volumetric reductions in medial/prefrontal regions and cingulate in adults and children with a history of maltreatment/trauma (e.g., physical neglect) [21–26]. These structures have been implicated in emotion regulation and memory processing and tend to exhibit morphometric and functional changes in MDD. Further, this research suggests that maltreatment/trauma is associated with structural modulations persisting into adulthood. To our knowledge, the interaction between age of MDD onset and trauma history on cortical thickness in depression has not been assessed.

As such, this pilot study examined cortical thickness in nonelderly adults (i.e., <60 years of age) with MDD to expand on the relatively scant and inconsistent literature on the matter. Second, we sought to assess whether differences existed in pediatric (<24 years of age) compared with adult MDD onset (>25 years of age) on cortical thickness, in an effort to replicate and expand on previous work. Third, we examined whether differences existed in cortical thickness in depressed adults with childhood sexual and/or physical abuse (sexual + physical abuse group—referred to simply as the abuse group) versus those who experienced no sexual and/or physical abuse but experienced emotional neglect/abuse and/or physical neglect (no sexual + physical abuse group (referred to simply as the non-abuse group); the abuse group also experienced emotional and physical neglect; Table 1). The interaction between age of MDD onset and trauma was also explored.

We expected thinner cortices in orbitofrontal, DLPFC, para-/postcentral, and insular cortices in MDD (versus controls) as well as greater reductions in the pediatric (versus adult) MDD onset group in the DLPFC and posterior inferior temporal regions relative to the adult onset group. Finally, we expected greater thinning in the MDD group with a history of childhood abuse in cortical regions comprising the frontal-limbic network. No directional hypotheses existed regarding age of MDD onset and trauma history due to lack of precedent literature.

## 2. Methods

### 2.1. Participants

Thirty-six adults (age range: 19–58 years) with a primary diagnosis of MDD were tested. Clinical diagnoses were made by the study psychiatrist (R.R.) according to the Structured Clinical Interview for DSM (Diagnostic and Statistical Manual of Mental Disorders) IV-TR Diagnoses, Axis I, Patient Version (SCID-IV-I/P) criteria. The Hamilton Rating Scale for Depression (HAMD_17_) was used to assess symptom severity [27], with patients being included if they had an HAMD_17_ score of ≥18. All participants were free of psychotropic medications for a minimum of three weeks at time of neuroimaging. Exclusion criteria included bipolar disorder (BP-I/II or NOS), psychosis history, a clinically significant anxiety disorder, current (<6 months) substance abuse/dependence, neurological disorders, eating disorders, unstable medical condition, and significant suicide risk. Participants with magnetic resonance imaging (MRI) contraindications (e.g., pregnancy, metal implants, and claustrophobia) were also excluded. Twenty patients had MDD onset at <24 years of age (pediatric onset) and 16 patients had MDD onset at >25 years of age (adult onset). Childhood traumatic events were assessed with the Childhood Trauma Questionnaire-Short Form (CTQ-SF) [28]. The CTQ-SF (referred to simply as the CTQ) consists of five subscales with five questions each (range: 1–5): emotional abuse, physical abuse, sexual abuse, emotional neglect, and physical neglect as well as a total score (CTQ_Total_). For this study, cut-off scores of a minimum of 8 on the physical abuse, physical neglect and sexual abuse subscales, 10 on the emotional abuse subscale, and 15 on the emotional neglect subscale were used. These thresholds are linked with moderate-to-severe levels of abuse and neglect [29]. MDD patients were divided into two groups based on early exposure to physical or sexual abuse: group 1 (abuse group): sexual and/or physical abuse (*N* = 12); group 2 (nonabuse group): no sexual and/or physical abuse (but presence of emotional neglect/abuse and/or physical neglect) (*N* = 19). Most patients who had a history of physical or sexual abuse also experienced some form of emotional maltreatment. Five MDD subjects did not complete the CTQ and were not included in the analyses pertaining to trauma.

Eighteen healthy controls (HCs) without any psychiatric history were also tested; HCs were not included in the cortical thickness analyses regarding trauma history. Informed consent was obtained prior to study initiation in compliance with the Conjoint Health Research Ethics Board at the University of Calgary. Participant characteristics are presented in Tables 1 and 2.

### 2.2. Magnetic Resonance Imaging (MRI): 3D Image Acquisition

Images were collected at the Seaman Family MR Centre (Foothills Hospital, University of Calgary) with a 3 T General Electric scanner (Signa LX, Waukesha, WI, USA) using a receive-only eight-channel RF head coil. A 3D T1-weighted magnetization prepared rapid acquisition gradient echo (MPRAGE) image was acquired (TR = 8.3 ms; TE = 1.8 ms; flip angle = 20°; voxel size = 0.5 × 0.5 × 1 mm; 1 mm slice thickness; 176 slices).

### 2.3. Cortical Thickness Analyses

Cortical thickness analyses were carried out using FreeSurfer software (http://surfer.nmr.mgh.harvard.edu/). Detailed procedures on cortical thickness analyses using FreeSurfer have been published [30–33]. In brief, T1-weighted images were intensity-normalized (correcting for magnetic field inconsistencies) and then a skull-stripping procedure was applied to remove extracerebral voxels. A researcher (F.M.-blind to identity/diagnoses) then carried out manual edits to the skull-stripped images. Scans subsequently underwent a segmentation procedure using an estimation of the structure of the grey-white interface. In order to create a smooth spherical representation of the grey-white interface and pial surface, each scan was covered with a triangular tessellation and inflated. Inflated scans were then aligned to FreeSurfer's default reference template via a 2D warp based on cortical folding patterns. Once smoothed using a circularly symmetric Gaussian kernel, sulci and gyri curvature patterns were aligned and the average cortical thickness was measured at each surface point. A uniform surface-based spherical coordinate system was created by transforming the reconstructed surfaces into parameterizable surfaces. An averaging procedure (50 iterations) was applied to smooth the surface and the reconstructed pial surface refined with a deformable surface algorithm. Data was again aligned on a common spherical coordinate system. Cortical thickness was determined by measuring and averaging the distance between the grey-white and pial surfaces [30–33].

### 2.4. Statistical Analyses

Groups were compared on demographic and clinical indices using one-way analyses of variance (ANOVAs). These analyses were first carried out between the MDD versus HC groups; subsequently, assessments were conducted with three levels (MDD, pediatric onset, adult onset) comprising the group variable. Clinical and demographic features were also compared with one-way ANOVAs between the pediatric and adult onset groups (Table 1).

A multivariate ANOVA (MANOVA) was carried out to assess cortical thickness differences across regions (see Table 1 in Supplementary Material available online at http://dx.doi.org/10.1155/2014/410472) between the MDD and HC groups. The MANOVA was followed by exploratory repeated-measures ANOVAs (rmANOVA; hemisphere as the within- and group (MDD, HC) as the between-subject factor) for each of regional cortical thickness measures (significance set at *P* < .01).

Subsequently, a multivariate analysis of covariance (MANCOVA) was carried out to assess cortical thickness measure differences across regions between the three groups (HC, pediatric onset, and adult onset); age was used as a covariate since it differed in the HC versus the adult and pediatric onset groups (Section 3.2). The MANCOVA was followed by exploratory rmANCOVAs (age as covariate; hemisphere as the within-subject factor; group (HC, pediatric onset, and adult onset) as the between-subject factor) assessing thickness in each cortical region; significance was set at *P* < .01.

One-way ANOVAs were carried out to compare the MDD groups with childhood abuse + neglect versus nonabuse + neglect (i.e., abuse and nonabuse groups, resp.) on pertinent demographic and clinical variables. A MANCOVA was carried out to assess cortical thickness measure differences across regions between the two groups (nonabuse, abuse); HAMD_17_ scores were used as a covariate as they differed between the abuse and nonabuse groups (Section 3.3). This was followed by exploratory rmANCOVAs (HAMD_17_ as covariate; hemisphere as the within-subject factor; group (abuse, non-abuse) as the between-subject factor) assessing thickness in each cortical region; significance was set at *P* < .01.

MANCOVAs (HAMD_17_ scores and age as covariates) were carried out with the two MDD onset (adult, pediatric) and two trauma groups (abuse, nonabuse) as independent variables on cortical thickness measures across regions. Exploratory rmANCOVAs (HAMD_17_ scores and age as covariates; hemisphere as within and groups as between-subject factors) were then carried out for thickness in each cortical region; significance was set at *P* < .01.

Finally, exploratory Spearman's correlations were carried out (for the MDD group) between CTQ_Total_ scores, abuse scores (physical + sexual abuse CTQ scores) and neglect scores (emotional abuse + emotional neglect + physical neglect CTQ scores), and all regional cortical thickness measures; significance was set at *P* < .005. Similarly, correlations were carried out between HAMD_17_ and all regional cortical thickness measures (MDD group only); significance was set at *P* < .005. Unless stated otherwise, means and standard deviations (SDs) are presented for all results. All cortical thickness measures are expressed as mm.

## 3. Results

### 3.1. Two Group Analyses (HC and MDD)

One-way ANOVAs (group (MDD and HCs) as the between-subject factor) revealed no main effect of group on age (Table 1).

**Figure 1:**
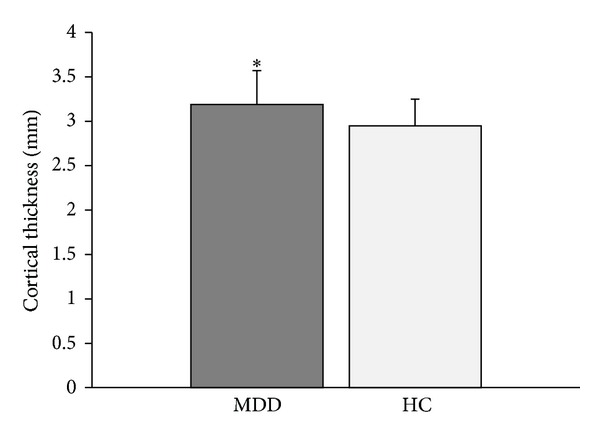
The group with major depressive disorder (MDD) had a thicker frontal pole compared with the healthy control (HC) group (*P = .01).

The MANOVA (group (HC, MDD) as fixed factor and regions as dependent variables) yielded no main effects of group on cortical thickness. In an effort to replicate previous research, the MANOVA was followed up by exploratory rmANOVA, with hemisphere as the within- and group (MDD, HC) as between-subject factors on cortical thickness (per region). Significance was set at *P* < .01 to minimize false positives and control for multiple comparisons. The main effects of hemisphere, as found by the rmANOVAs, on cortical thickness in various brain regions are listed in Table 3. The rmANOVA revealed a main effect of group (MDD, HC) on frontal pole thickness (*F*[1,52] = 7.05, *P* = .01), with a thicker cortex in the MDD (3.19 ± .38) versus the HC group (2.95 ± .30; Figure 1). A trend for a main effect of group was noted on transverse temporal thickness (*F*[1,52] = 6.49, *P* = .014), with a thinner cortex in the MDD (3.12 ± .17) versus the HC group (3.22 ± .15).

### 3.2. Three Group Analyses (Adult MDD Onset, Pediatric MDD Onset, and HCs) and Two Group Analyses (Adult and Pediatric MDD Onset)

One-way ANOVAs were carried out with group as the independent variable (3 groups: pediatric onset: onset <24 yrs; adult onset: onset >25 yrs; HCs) and age as the dependent variable. A main effect of group existed (*F*[2,51] = 9.97, *P* < .001); follow-up comparisons indicated a difference between the adult MDD onset and both the HC (*P* < .001) and pediatric MDD onset groups (*P* < .001), with the adult onset group being the oldest (Table 1).

Further one-way ANOVAs were carried out between the pediatric versus adult MDD onset groups on other pertinent variables (i.e., HAMD_17_ scores, duration of current MDE, and time since diagnoses). A main effect of group was noted for HAMD_17_ scores (*F*[1,34] = 6.50, *P* = .015), with higher scores in the adult verses the pediatric MDD onset group.

The MANCOVA (age as a covariate) yielded no main effect of group (3 groups: HC, adult onset, and pediatric onset) on cortical thickness. However, given the pilot nature of this work, the MANCOVA was followed up with exploratory rmANCOVAs (age as a covariate; hemisphere as the within- and group as the between-subject factor) assessing thickness in each cortical region. Significance was set at *P* < .01. A main effect of hemisphere was noted on cortical thickness in the rostral middle frontal cortex (*F*[1,50] = 7.38, *P* = .009; right > left). A trend for a main effect of group (3 groups) on frontal pole cortex thickness was noted (*F*[2,50] = 4.64, *P* = .014), with a thinner cortex in the HC group (2.93 ± .30) versus the pediatric MDD onset group (3.22 ± .39; *P* = .005).

### 3.3. Two Group Analyses (MDD Groups: Childhood Abuse Group and Nonabuse Group)

One-way ANOVAs were conducted to compare the childhood abuse (*N* = 12) versus non-abuse (*N* = 19) groups on pertinent demographic and clinical variables (i.e., time since MDD diagnosis, HAMD_17_ scores, current age, age of MDD onset, and duration of current MDE). A main effect of group (neglect, abuse) was found on HAMD_17_ scores (*F*[1,29] = 4.21, *P* = .049), with higher scores in the abuse group. The abuse group also had higher CTQ_Total_ (*F*[1,29] = 27.38, *P* < .001) and, expectedly, abuse scores (*F*[1,29] = 42.04, *P* < .001) than the nonabuse group (Table 2).

The MANCOVA, with group (nonabuse, abuse) as the independent variable, was carried out on cortical thickness measures (HAMD_17_ scores were the covariate)—no main group effect on cortical thickness existed. Exploratory rmANOVAs (group as between- and hemisphere as within-subject factors, HAMD_17_ as the covariate) yielded no significant results, apart from a weak trend for a main effect of group (*F*[1,28] = 3.26, *P* = .082) on frontal pole cortical thickness. This trend was followed up with univariate ANOVAs assessing frontal pole thickness in each hemisphere (HAMD_17_ as covariate). A trend for main effect of group on right frontal pole cortex thickness (*F*[1,28] = 4.20, *P* = .05) was found, with a thicker cortex in the abuse (3.36 ± .37) versus the nonabuse group (3.10 ± .25).

An inverse correlation was found between left precuneus cortex thickness (*r* = − .57, *P* < .001, *N* = 31) as well as right middle temporal cortex thickness (*r* = −.59, *P* < .001, *N* = 31) and CTQ_Total_ scores. Similarly, an inverse correlation existed between both left (*r* = − .51, *P* = .003, *N* = 31) and right (*r* = − .54, *P* = .002, *N* = 31) frontal pole cortex thickness and CTQ_Total_ scores. An inverse correlation also existed between right frontal pole cortex thickness and “abuse” scores (*r* = − .50, *P* = .004, *N* = 31). An inverse relation existed between left inferior parietal cortex thickness (−.59, *P* < .001, *N* = 31) as well as right superior parietal cortex thickness (−.53, *P* = .002, *N* = 31) and “neglect” scores.

Finally, Chi-square tests revealed no significant difference in the proportion of the pediatric versus adult MDD onset individuals in either the abuse or nonabuse groups. MANCOVAs (HAMD_17_ scores and age as covariates) were carried out with the two MDD onset (adult and pediatric) and two childhood trauma groups (abuse and nonabuse) as the independent variables on cortical thickness measures. No main group effects or interactions were found. Exploratory rmANOVAs (HAMD_17_ scores and age as covariates; hemisphere as with- and groups as between-subject factors; significance was set at *P* < .01) yielded a trend for an onset group × childhood trauma group interaction for superior temporal cortex thickness (*F*[1,25] = 5.98, *P* = .022), with pairwise comparisons indicating a trend for a difference in cortical thickness between the pediatric (*N* = 8; 2.59 ± .14) and adult (*N* = 8; 2.77 ± .14) MDD onset groups with childhood abuse (*P* = .02). For frontal pole thickness, an onset group × childhood trauma group × hemisphere interaction trend existed (*F*[1,25] = 5.07, *P* = .033). Pairwise comparisons indicated a trend for a difference (*P* = .026) in right frontal pole cortical thickness between the abuse (*N* = 8; 3.01 ± .15) and nonabuse (*N* = 8; 3.42 ± .41) groups in the adult MDD onset cohort.

## 4. Discussion

In brief, this pilot study aimed to contribute to existing literature on cortical thickness in depressed adults in two ways. First, we sought to clarify the effect of age of MDD onset on spatial cortical thickness patterns. Second, we investigated the role of childhood trauma, in the form of abuse or nonabuse history (though both groups experienced neglect), on cortical thickness in MDD and its interaction with age of disorder onset. We found thicker frontal pole cortices in the MDD versus HC group. Conversely, a tendency for a thinner transverse temporal cortex existed in MDD. With respect to age of onset, clinically, the adult versus pediatric onset group exhibited higher HAMD_17_ scores. The pediatric onset group had a thicker frontal pole cortex than HCs. In comparisons of MDD groups with childhood abuse versus nonabuse history (the abused group also exhibited neglect and had higher CTQ_Total_ scores), the abuse group had greater HAMD_17_ scores. A tendency for a thicker cortex was noted in the abuse versus nonabuse group in the right frontal pole. Inverse correlations existed between the left precuneus, right middle temporal as well as bilateral frontal pole cortical thickness, and CTQ_Total_ scores. Inverse relations were also noted between right frontal pole cortex thickness and CTQ abuse scores as well as between the left inferior and right superior parietal cortex thickness and CTQ neglect scores. Finally, the superior temporal cortex tended to be thinner in the pediatric versus adult onset groups with childhood abuse. Additionally, the right frontal pole cortex tended to be thinner in the abuse versus nonabuse groups in the adult onset group.

The role of the frontal poles in MDD (and outside the context of the disorder) is not well understood. Neuroimaging studies suggest that frontal poles play a role in “cognitive branching” (i.e., flexibility) as they are activated when performing several subgoals while keeping in mind another (main) goal. Though a handful of functional MRI (fMRI) studies have implicated frontal pole activity in response to antidepressant interventions [34, 35], few morphometric studies of the frontal poles in MDD exist. Much of the work linking the frontal poles with depression stems from stroke research, where greater depression severity has been associated with increased lesion proximity to the frontal poles [36].

Unlike Sheline et al. [7], we noted thicker frontal pole cortices in MDD versus HCs. However, since their sample consisted of late-life depressed individuals while ours was comprised of relatively young-to-middle aged adults, the results may not be directly comparable. They also found that thinner frontal pole cortices existed in patients who did not achieve remission compared to those who did. Given that greater frontal pole cortical thickness in our study was driven by the pediatric onset group, it is feasible that these individuals may have been more likely to be treatment responders (versus the adult onset group). However, as response was not assessed in the current study (though this represents a worthy future direction), this interpretation is speculative. Further, because the pediatric onset group was characterized by lower HAMD_17_ scores than the adult onset group, thicker frontal pole cortices may reflect a neurocompensatory/adaptive mechanism in the disorder. Neurocompensatory mechanisms are more likely during adolescence, which is a period associated with extensive brain plasticity [14–16]. Additionally, the right frontal pole cortex tended to be thinner in the abuse versus nonabuse groups in the adult MDD onset group suggesting that more pronounced trauma might make the brain susceptible to the neural consequences associated with a psychiatric condition in adulthood.

Few studies have assessed (or reported on) the significance of the transverse temporal cortex (Heschl's gyrus) in MDD. One fMRI study found greater right Heschl's gyrus activation during an emotive processing/attention control task in individuals with a family history of depression versus those without a family history [37], suggesting that the region may play some role in MDD. However, postmortem examinations yielded no differences in neural or glial cell density or cortical thickness in the Heschl's gyrus between individuals with MDD and HCs [38]. Another group found volumetric reductions in the superior temporal gyrus (not Heschl's gyrus specifically) in recovered depressed participants [39], which is somewhat consistent with our observed trend for a thinner transverse temporal cortex in MDD. We also noted a tendency for a thinner superior temporal cortex in the pediatric versus adult onset groups with childhood abuse suggesting that abuse during critical neurodevelopmental periods may influence cytoarchitecture within this region.

Childhood maltreatment is strongly associated with increased risk for psychiatric disorder development [22]. By extension, neural abnormalities associated with trauma/maltreatment may increase psychiatric disorder vulnerability. Previous work has reported reduced cortical thickness and volume in maltreated versus nonmaltreated children in the anterior cingulate, superior frontal gyrus, and orbitofrontal cortex [19, 25]. Similarly, childhood emotional maltreatment and physical neglect were associated with reductions in medial prefrontal cortex volumes in adults [21, 22]. Nondepressed subjects with a family history of MDD and a history of emotional abuse exhibited smaller DLPFC, medial prefrontal, and anterior cingulate cortices than controls [23]. Yet another group found that decreased cingulate volume in individuals with MDD was related to abuse history [5]. Finally, Dannlowski et al. [24] reported reduced grey matter volumes in regions including the orbitofrontal cortex and anterior cingulate gyrus in adults with high CTQ scores. The above indicates that prefrontal, anterior cingulate cortex, and lateral temporal regions (areas implicated in MDD) tend to be rather consistently affected by maltreatment/trauma. Further, research suggests that maltreatment/trauma is associated with structural damage that persists into adulthood. These results mimic our findings of an inverse relation between cortical thickness in the frontal poles, precuneus, and middle temporal regions and CTQ_Total_ scores as well as inverse relations between abuse scores and left inferior and right superior parietal cortical thickness.

The primary limitation of this study was its exploratory nature as well as the small sample size, specifically when groups were split by abuse/nonabuse history. Assessments of interactions between age of MDD onset and childhood trauma (i.e., 2 × 2 group comparisons) on cortical thickness—though highly novel—were statistically underpowered. Further, in an effort to correct for multiple comparisons and decrease false positive rates, we included covariates when appropriate; inclusion of covariates further decreases power. Due to these limitations, it was not feasible to meaningfully explore the influence of sex on cortical thickness in this study, which may have been informative. In a similar vein, although we attempted to correct for multiple comparisons by adjusting our significance level, true corrections (e.g., Bonferroni) were not applied, though this should be done in comparable future work. As such, our findings and conclusions should be treated as preliminary and with caution, warranting further replication and expansion with a larger sample size.

Briefly, the focal future direction of this work is to assess cortical thickness in a large sample of well-characterized depressed individuals in terms of their trauma history and MDD onset age in order to disambiguate the contributions of these factors in influencing cortical cytoarchitecture in MDD. Greater clarity is needed to better understand the multiple, likely interacting, factors that contribute to altered cortical thickness patterns in MDD. Assessments of such well-characterized samples over time (versus cross-sectionally) would also allow us to gain better insight regarding the neurodevelopmental processes across the lifespan in the context of depression.

## Supplementary Material

The regions outlined in Supplementary Table 1 were assessed in the cortical thickness analyses in both hemispheres.

## Figures and Tables

**Table 1:** Characteristics of MDD onset groups (pediatric/adult MDD onset) and controls.

Characteristics	MDD (overall)	Pediatric MDD onset	Adult MDD onset	HC
*N*	36	20	16	18
Sex (F/M)	22/14	12/8	10/6	10/8
Age (yrs.)	37.1 ± 11.2	31.5 ± 10.5	44.1 ± 7.7	31.9 ± 9.2
Baseline HAMD_17 _	22.1 ± 4.1	20.7 ± 4.1	23.9 ± 3.4	—
Duration of current MDE (yrs.)	5.1 ± 5.4	4.9 ± 5.7	5.4 ± 5.3	—
Time since MDD onset (yrs.)	12.3 ± 9.2	14.4 ± 10.7	9.8 ± 6.3	—
MDD onset (yrs.)	24.8 ± 10.1	17.1 ± 4.8	34.3 ± 5.6	—

HC: healthy controls; HAMD_17_: Hamilton Depression Rating Scale; MDD: major depressive disorder; MDE: major depressive episode; means ± SDs presented.

**Table 2:** Characteristics of childhood abuse and nonabuse MDD groups.

Characteristics	Nonabuse MDD group	Abuse MDD group
*N*	19	12
Sex (F/M)	10/9	8/4
Age (yrs.)	36.4 ± 12.6	40.0 ± 9.5
Baseline HAMD_17 _	21.2 ± 4.0	24.7 ± 3.9
Duration of current MDE (yrs.)	4.7 ± 5.2	5.4 ± 6.1
Time since MDD onset (yrs.)	11.1 ± 9.4	11.9 ± 9.1
MDD onset (yrs.)	25.3 ± 11.1	28.1 ± 7.3
CTQ_Total_	51.0 ± 6.1	62.9 ± 11.1
CTQ “neglect” score	39.8 ± 5.6	42.5 ± 6.5
CTQ “abuse” score	11.2 ± 1.1	21.3 ± 6.7

CTQ: Childhood Trauma Questionnaire; CTQ “neglect” score: emotional neglect + physical neglect + emotional abuse; CTQ “abuse” score: physical abuse + sexual abuse; HAMD_17_: Hamilton Depression Rating Scale; MDD: major depressive disorder; MDE: major depressive episode; means ± SDs presented.

**Table 3:** Cortical thickness hemispheric differences.

Region (cortical thickness)	Hemisphere effect	*P* value
Caudal middle frontal cortex	L > R	.003
Entorhinal cortex	L > R	.001
Fusiform cortex	L > R	<.001
Inferior parietal cortex	L > R	.005
Inferior temporal cortex	L > R	<.001
Isthmus cingulate cortex	L > R	<.001
Lingual cortex	R > L	.005
Pars orbitalis cortex	R > L	<.001
Pericalcarine cortex	L > R	.006
Precentral cortex	L > R	.003
Precuneus cortex	R > L	.001
Rostral anterior cingulate cortex	R > L	<.001
Rostral middle frontal cortex	R > L	<.001
Superior frontal cortex	L > R	<.001
Superior parietal cortex	R > L	.001
Superior temporal cortex	L > R	<.001

L: left hemisphere; R: right hemisphere.
