# Supplementary material for: A Preliminary Study of the Influence of Age of Onset and Childhood Trauma on Cortical Thickness in Major Depressive Disorder
Source: Biomed Res Int. 2014 Mar 6;2014:410472. doi: 10.1155/2014/410472 (PMC3966405; doi:10.1155/2014/410472)
Supplement: Supplementary file 1 — The regions outlined in Supplementary Table 1 were assessed in the cortical thickness analyses in both hemispheres. [file 410472.f1.docx]

**Supplementary Table 1.** Regions assessed in the cortical thickness analyses (both hemispheres)

| caudal anterior cingulate cortex | parstriangularis cortex |
| --- | --- |
| caudal middle frontal cortex | pericalcarine cortex |
| cuneus cortex | postcentral cortex |
| entorhinal cortex | posterior cingulate cortex |
| fusiform cortex | precentral cortex |
| inferiorparietal cortex | precuneus cortex |
| inferiortemporal cortex | rostral anterior cingulate cortex |
| isthmus-cingulate cortex | rostral middle frontal cortex |
| lateralorbitofrontal cortex | superior frontal cortex |
| lingual cortex | superior parietal cortex |
| medial orbitofrontal cortex | superior temporal cortex |
| middle temporal cortex | supramarginal cortex |
| parahippocampal cortex | frontal pole cortex |
| paracentral cortex | temporal pole cortex |
| parsopercularis cortex | transverse temporal cortex |
| parsorbitalis cortex |  |
